# Structural Modelling Prediction of Recombinant *Plasmodium falciparum* K13-F446I and K13-C580Y Gene by AlphaFold Method and Heterologous Expression in *Spodoptera frugiperda* 9 Cells

**DOI:** 10.3390/pathogens11111271

**Published:** 2022-10-31

**Authors:** He Yan, Jun Feng, Min Chen

**Affiliations:** 1 National Institute of Parasitic Diseases, Chinese Center for Disease Control and Prevention (Chinese Center for Tropical Diseases Research), NHC Key Laboratory of Parasite and Vector Biology, WHO Collaborating Centre for Tropical Diseases, National Center for International Research on Tropical Diseases, Shanghai 200025, China; 2 Shanghai Municipal Center for Diseases Control and Prevention, Shanghai, 200336, China

**Keywords:** *Plasmodium falciparum*, Kelch 13 protein, F446I, C580Y, baculovirus, eukaryotic expression

## Abstract

*P. falciparum* Kelch 13 (Pfk13) is an essential protein that contains BTB and Kelch-repeat propeller domains (KRPD), which was predicted to bind substrate during ubiquitin-dependent degradation pathway. However, the function of Pfk13 and the structural alterations associated with artemisinin resistance mutations remain unknown. Herein, we screened two proteins, namely Pfk13-F446I and Pfk13-C580Y, which are closely associated with artemisinin, for structural prediction analysis. The 389 amino acids from 1011 nt to 2178 nt of KRPD were cloned into pFastBac^TM^1. The recombinant plasmids were heterologously expressed in *Spodoptera frugiperda* 9 cells (SF9) and a ~44 kDa protein band was yielded by SDS-PAGE and Western Blot. A total of five structure models were generated and predicted by AlphaFold for each protein. The models predicted that Pfk13-F446I would be located in the central protein cavity, proximal to mutations in cysteine residues primarily in β strands. Unlike Pfk13-F446I, the Pfk13-C580Y is located on the small channel that runs through the center of the K13 protein. Interestingly, the hydrogen bond between C580 and C533 in the wide type (WT) was not detected, suggesting that the hydrogen bond may be lost during the mutation. Besides, the Pfk13-F446I and Pfk13-C580Y mutation were found to add 11 and 9 hydrogen bonds variations that may lead to conformational change of the protein structure compared to WT, respectively. Future work should pay more attention to the binding characteristics of those mutations related with KPRD pockets and their binding substrates, which will further clarify the structure and function of Pfk13 and its mutant.

## 1. Introduction

Malaria is one of the most severe public health issues worldwide. Among all five *Plasmodium* species that infect humans, *Plasmodium falciparum* has been the deadliest parasite [1]. Currently, malaria burden has greatly declined due to the implementations of artemisinin (ART)-based combination therapies (ACTs), recommended as the first line drug by the World Health Organization (WHO) in endemic countries [2]. However, treatment failure has been seen in some regions due to the emergence and spread of artemisinin resistance against *P. falciparum* at the Thai-Cambodia border in the Greater Mekong subregion (GMS), which imperils the global effort to reduce the burden of malaria [3,4]. ART resistance (ART-R) is defined as in vivo delayed parasite clearance following treatment with an artesunate monotherapy or with an ACT, and in vitro, an increased survival rate following exposure to a high dose of ART [5,6]. This is primarily conferred by the *P. falciparum* Kelch 13 (*Pfk13*) locus, identified as a genetic marker for ART-R by sequencing of ART-pressured parasite strains in vitro and ART-sensitive or -resistant Cambodian isolates [7]. The Pfk13 protein is a member of the Kelch-like superfamily and is dimeric as per its crystal structure [8,9,10]. Till now, 10 of the *Pfk13* single nonsynonymous mutations have been validated in vitro and in vivo as associated with delayed clearance following ACT treatment [11]. Among them, C580Y, assumed to be one of the strong molecular markers for ART-R in *P. falciparum*, was predominant across GMS including Cambodia, Myanmar, Thailand, Laos, and Vietnam [12,13]; it was also reported in the Amazonia region and Africa [14,15]. Another ART-R-related *Pfk13* mutation, F446I, was commonly observed at the China-Myanmar border and in northern Myanmar [16,17]. Previous studies investigated the increased survival rate of RSA_0–3h_ by the re-transfected F446I gene into the *P. falciparum* 3D7 strain, which was significantly associated with day three parasitemia in patients [18]. The transgenic experiment showed that parasite carrying the F446I mutation displayed prolonged clearance in response to ART, while using C580Y as a positive control mutant [13]. Therefore, identification of novel drug targets and scaffolds to cover the eventual emergency of complete ACT failure has prompted global concern. Drug repurposing methodologies and structure-based conservation techniques that target invariant parasite house-keeping proteins may lead to novel foci for drug development [19]. The structure of the Pfk13 protein has previously been analyzed for the distribution of ART-R mutations and its dimerization interfaces, utilizing the publicly available three-dimensional structure [20,21]. To date, two crystal structures of Pfk13 proteins are available in RCSB Protein Data Bank (4YY8 and 4ZGC). The protein structures showed that they are clustered in two specific regions–one is the surface-exposed residues implicated in protein-protein interactions, and the other is the buried residues influencing the overall Pfk13 structure [22]. In this study, we aim to provide a heterologous expression system using the baculovirus expression vector system (BEVS) for Pfk13-F446I and Pfk13-C580Y, into the *Spodoptera frugiperda* 9 cell (SF9), and to investigate and characterize the structural modelling analysis of the two mutated proteins with the AlphaFold method, which may provide some novel insights into the contributions of those two key sites with ART-R in Pfk13.

## 2. Materials and Methods

### 2.1. Strains, Insect Cells and Materials

The DH10Bac strain, *Spodoptera frugiperda* 9 cells (SF9) and pFastBac^TM^1 transfer plasmid were all purchased from Shanghai NovoPro Biotechnology Co., Ltd (Shanghai, China). SF9 cells used in this study were grown at 28 °C in sterile shake flasks at a speed of 140 revolutions per minute (rpm) in a shaking incubator.

### 2.2. Chemosynthesis, Construction and Verification of Recombinant Plasmids with Pfk13-WT-Bac, Pfk13-F446I-Bac, and Pfk13-C580Y-Bac

The total 389 amino acids sequence from 1011 nt to 2178 nt of KRPD in Kelch 13 (PF3D7_1343700) related to the mutation of *P. falciparum* 3D7 were obtained from the PDB website (http://www.rcsb.org/structure/4YY8; accessed on 10 September 2021). Then, the nucleobase sequence of WT, Pfk13-F446I (Phe to Iso, TTT change into ATT) and Pfk13-C580Y (Cys to Tyr, TGT change into TAT) with the enzymatic sites of *BamH*I (GGATCC) and *EcoR*I (GAATTC), respectively, underwent chemosynthesis by Shanghai NovoPro Biotechnology Co., Ltd. The above fragments were then ligated with the expression vector pFastBac^TM^1 to obtain Pfk13-WT-Bac, Pfk13-F446I-Bac and Pfk13-C580Y-Bac. The recombinant plasmid was sequenced to confirm the insert sequence; it was also confirmed by double enzymatic assay with *BsrG*I and *Hind*III. After that, the recombinant plasmid was transformed into DH10Bac competent cells and coated with tertiary antibiotics (50 μg/mL kanamycin; 7 μg/mL gentamicin; 10 μg/mL tetracycline). Incubated plates with X-gal (final concentration of 1 mM) and isopropyl-β-d-thiogalactopyranoside (IPTG) (final concentration of 0.1 mM) were incubated overnight at 37 °C. The white colonies were selected and inoculated into 5 mL tertiary antibiotics (50 μg/mL kanamycin; 7 μg/mL gentamicin; 10 μg/mL tetracycline) LB medium and cultured overnight at 37 °C at 220 rpm. A small number of plasmids were extracted and verified by enzymatic digestion using PCR. 

### 2.3. Transfection and Collection of SF9 Cell with Pfk13-WT, Pfk13-F446I and Pfk13-C580Y

A total of 2 mL of SF9 cells with a density of 0.5 × 10^6^/mL were inoculated into a six-well plate and adherent growth at 27 °C. The plasmids Pfk13-WT, Pfk13-F446I and Pfk13-C580Y were fully reacted with the transfection reagent, and then gently added to SF9 cells. The cells were incubated at 27 °C until they showed signs of virus infection. The remaining culture was centrifuged and transferred to a sterile 2.0 mL EP tube, stored at 4 °C and protected from light. 

### 2.4. In Vitro Purification of Recombinant Protein of Pfk13-WT, Pfk13-F446I and Pfk13-C580Y

To obtain the purified protein, a total of 30 mL of SF9 cells with Pfk13-WT, Pfk13-F446I and Pfk13-C580Y at a density of 2.0 × 10^6^/mL were incubated into a 250 mL Erlenmeyer flask and cultured at 27 °C and at 120 rpm for 4 days. After addition of IPTG at 0.2 mM, the broth was induced at 16 °C for 20–24 h. Cells were harvested by centrifuge at 3996× *g* for 10 min. The cells were resuspended in PBS (pH 7.4 with 1 mM PMSF), quickly frozen in liquid nitrogen, and stored at −80 °C. After thawing, NP-40 (0.6%), the protease inhibitor, and DNase I were added, pulverized by ultrasonic sound waves at 4 °C (5 s on/7 s off, 5 min, 100 W), and centrifuged at 63,936× *g* at 10 °C for 30 min. Then the precipitation of recombinant protein was collected and used for assay. The recombinant proteins with His-tag were purified with Ni-NTA agarose (Qiagen, Germantown, MD, USA). After the cells were lysed, the supernatant was loaded at a rate of 1–1.5 mL/min and was allowed to load onto a Bio-Rad 10-DG desalting column (GE Healthcare, Marlborough, MA, USA), which was pre-equilibrated with 50 mM HEPES buffer (pH 7.5) and with 5% (*v*/*v*) glycerol. The protein elution (50 mM HEPES, 500 mM NaCl, 5% Glycerol, pH 7.5) was adopted with gradient elution (from 5 to 250 mM Imidazole) and was eluted at 250 mM Imidazole; then 1 mM TCEP was added to the eluted sample. The eluted sample was dialyzed into buffer (50 mM HEPES, 500 mM NaCl, 5% Glycerol, pH 7.5). The final protein was dialyzed into buffer (10 mM HEPES, 500 mM NaCl, 5% Glycerol, pH 7.5) and then aliquoted. 

### 2.5. Western Blot

The recombinant protein expression was verified by western blot (WB) assay. Briefly, the extracted proteins were denatured by boiling with SDS-PAGE buffer, separated by 12% SDS-PAGE, and transferred to polyvinylidene difluoride membranes (Millipore, Burlington, MA, USA). The blots were blocked with a buffer containing 5% skimmed milk for 1 h at 25 °C, and incubated in the same buffer with rabbit anti-His antibodies (1:1000 dilution in blocking buffer, Merck, Darmstadt, Germany) overnight at 4 °C. After washing, the precipitation, supernatant, and elution (each for 20 μL) of recombinant protein was performed by incubation with appropriate secondary antibodies (goat anti-rabbit IgG conjugated to HRP at a dilution of 1:20,000, Merck, Darmstadt, Germany) for 1 h at room temperature. The blots were washed, and a signal was developed using the SuperSignal West Pico or Femto Chemiluminescent kit (Termo Fisher Scientifc, Waltham, MA, USA), and recorded on the BioRad ChemiDoc MP imaging system.

### 2.6. Protein Concentration Determination 

Recombinant Pfk13-WT, Pfk13-F446I and Pfk13-C580Y proteins contain no tryptophan residue, and UV at A_280_ was not ideal for protein determination. Protein concentration was determined by Bradford assay using bovine serum albumin (BSA) as a standard. The BSA assay kit (Sigma-Aldrich, Taufkirchen, Germany) was used according to manufacturer’s instructions. The purity of each protein was determined by BandScan Version 5.0.

### 2.7. Sequence Analysis

Protein comparison was carried out by protein BLAST tool (http://www.ncbi.nlm.nih.gov/blast/; accessed on 5 December 2021). Multiple amino acid sequence alignment was performed by Cobalt Constraint-based Multiple Alignment Tool (http://www.ncbi.nlm.nih.gov/tools/cobalt/cobalt.cgi?link_loc=BlastHomeLink; accessed on 12 May 2021.

### 2.8. AlphaFold Modelling Analysis 

The modelling of recombinant protein of Pfk13-WT, Pfk13-F446I and Pfk13-C580Y structure was conducted by AlphaFold (https://deepmind.com/research/open-source; accessed on 24 September 21). The structure prediction was performed by the standard AlphaFold pipeline. For further analysis, structures with the best prediction quality were selected and named the best-lowest model according to a predicted local distance difference test (pLDDT). Preparation of molecular graphics images and minimal distance measurements between atoms of pfK13-F446I and pfK13-C580Y were carried out in PyMol version 2.3.0 (Schrödinger, New York, NY, USA). 

### 2.9. Code Availability

Source code for the AlphaFold model, trained weights, and inference script are available under an open-source license at https://github.com/deepmind/alphafold; accessed on 5 December 2021.

## 3. Results

### 3.1. Constuction of Recombinant Pfk13-WT, Pfk13-F446I and Pfk13-C580Y 

**Figure 1:**
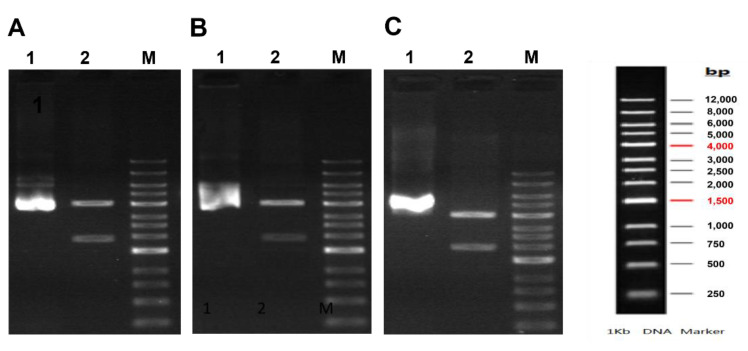
PCR assay of recombinant Bacmid DNA. (**A**) Pfk13-WT recombinant Bacmid DNA, (**B**) Pfk13-F446I recombinant Bacmid DNA, (**C**) Pfk13-C580Y recombinant Bacmid DNA. M: DL5000 Marker; 1: the recombinant plasmid, 2: the recombinant plasmid digested with *BsrG*I-*Hind*III.

The recombinant Pfk13-WT-Bac, Pfk13-F446I-Bac, and Pfk13-C580Y-Bac were constructed by using the 389 amino acids sequence from 1011 nt to 2178 nt of KRPD in pfK13. PCR assay indicated that the presence of a 4032 bp band and 1970 bp band by double enzymatic sites with *BsrG*I and *Hind*III for Pfk13-WT-Bac (Figure 1A), Pfk13-F446I-Bac (Figure 1B), and Pfk13-C580Y-Bac (Figure 1C). 

### 3.2. Expression of Pfk13-WT, Pfk13-F446I and Pfk13-C580Y in SF9 Cell

**Figure 2:**
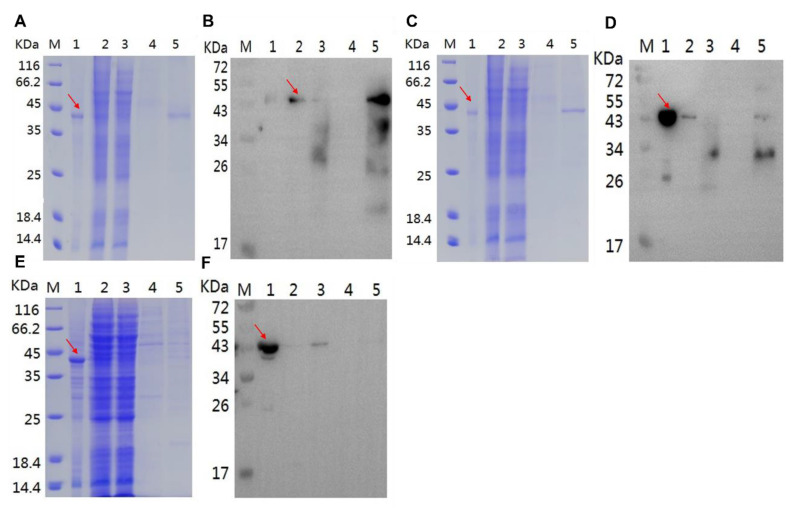
SDS-PAGE and WB assay of recombinant protein. (**A**,**B**) Pfk13-WT recombinant protein, (**C**,**D**) Pfk13-F446I recombinant protein, (**E**,**F**) Pfk13-C580Y recombinant protein. M: protein marker; 1: precipitation; 2: supernatant; 3: flow through; 4: wash; 5: elution. The red arrow indicates the expected protein location.

After transfected into SF9 cell, SDS-PAGE assay showed that Pfk13-WT (Figure 2A), Pfk13-F446I (Figure 2C) and Pfk13-C580Y (Figure 2E) proteins have been expressed in the SF9 virus system. The target bands of Pfk13-WT, Pfk13-F446I and Pfk13-C580Y were obtained with the purity of 91.2%, 94.5% and 82.4% in the supernatant, respectively. The target purified proteins of Pfk13-WT, Pfk13-F446I and Pfk13-C580Y were obtained and verified as ~44 kDa after dialysis. In addition, WB confirmed the presence of recombinant proteins of Pfk13-WT (Figure 2B), Pfk13-F446I (Figure 2D) and Pfk13-C580Y (Figure 2F). 

### 3.3. Structural Modelling of Pfk13-F446I and Pfk13-C580Y

**Figure 3:**
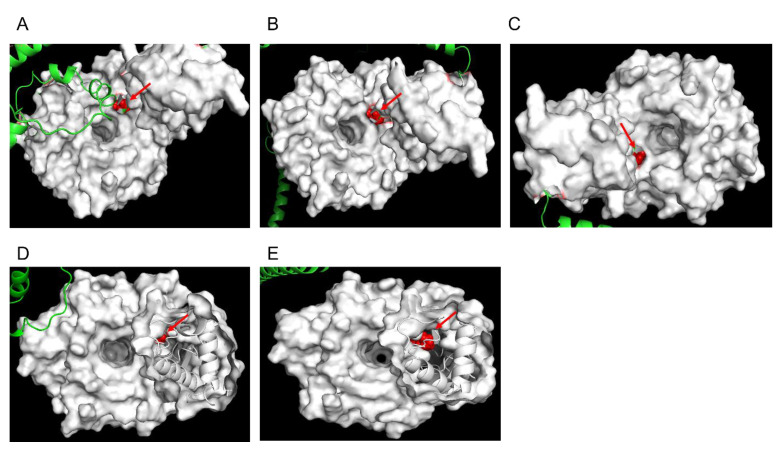
F446I protein structure modelling by AlphaFold. The structure prediction was performed by the standard AlphaFold pipeline and generates 5 structure models from the best-lowest model. The orthogonal views were listed in (**A**–**E**). The red arrow indicates the mutation at 446 site (change from Phe to Iso).

**Figure 4:**
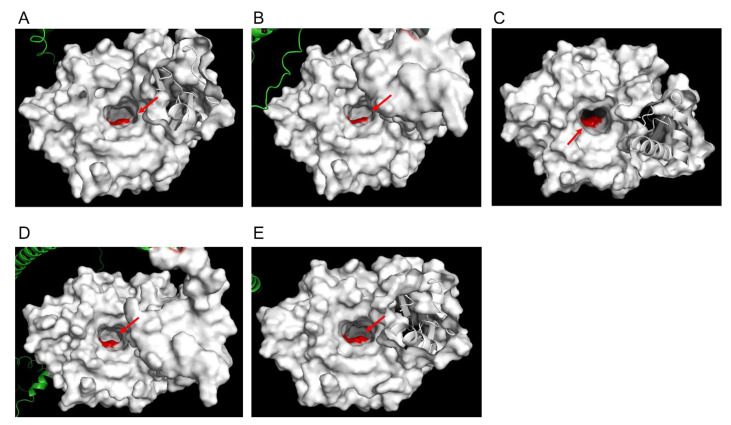
C580Y protein structure modelling by AlphaFold. The structure prediction was performed by the standard AlphaFold pipeline, and generated 5 structure models from the best-lowest model. The orthogonal views were listed in (**A**–**E**). The red arrow indicates the mutation at 580 site (change from Cys to Tyr).

To evaluate the structural characteristics of Pfk13-F446I and Pfk13-C580Y, we have conducted structural modelling using the AlphaFold method. From the best-lowest model (the pLDDT ranged from 85.436 to 92.654 for Pfk13-F446I, and from 82.115 to 93.748 for Pfk13-C580Y), a total of five structure models were generated each for Pfk13-F446I and Pfk13-C580Y, respectively. The structure of Pfk13 suggests a redox function owing to the presence of seven cysteine residues in the β-propeller domain. All five models showed that Pfk13-F446I are in the central protein cavity, proximal to mutations in cysteine residues primarily in β strands (Figure 3A–E). Unlike Pfk13-F446I, the Pfk13-C580Y are located on the small channel that runs through the center of K13 protein (Figure 4A–E). The amino acid replacements, structural variations of Pfk13-F446I (Table S1) and Pfk13-C580Y (Table S2) compared with the 3D model of Pfk13 from the Research Collaboratory for Structural Bioinformatics Protein Data Bank (RCSB PDB; ID: 4YY8) were analyzed, and the F446I and C580Y mutations added 11 and 9 hydrogen bonds variation when compared to WT, respectively. Interestingly, the hydrogen bond between C580 and C533 in the WT of PDB 4YY8 was not detected in our model, which suggests that the hydrogen bond may have been lost during the mutation (Table 1).

## 4. Discussion

The resistance of *P. falciparum* to ACTs is one of the major challenges facing malaria elimination worldwide, including China, where malaria had been eliminated in 2021 [23,24]. The ART-R of *P. falciparum* isolates have been found in five countries in the GMS, and genes with single nucleotide polymorphisms (SNPs) related to artemisinin resistance have been found in *P. falciparum* cases in Africa [17,25,26]. Since the first discovery linking Pfk13 to ART-R reported in 2014, little published literature has reported on the recombinant expression of Pfk13 protein using eukaryotic expression systems, except for the *E. coli* expression system [27]. In the study, we report for the first time the expression of Pfk13-F446I and Pfk13-C580Y proteins using BEVS and their structure modelling evaluation by the AlphaFold method. BEVS is a highly efficient eukaryotic expression system with high expression efficiency, similarity between expression, and natural products, etc. At present, it has been used in malaria transmission-blocking vaccines such as *P. falciparum* gametocyte surface protein pfs48/45 [28], and the mosquito midgut gene [29,30]. 

Previous bioinformatics studies have indicated that the Pfk13 protein is highly homologous to the human Kelch-like ECH-associated protein-1 (KEAP1). Therefore, it is proposed that K13 and KEAP1 proteins have similar functions through the regulation of the transcription factor NFE2-related factor 2 (nuclear factor-erythroid 2-related factor 2, Nrf2) ubiquitination process, which is the main regulator of oxidative stress [31]. The Pfk13 protein is a 726 amino-acid member of the Kelch-like (KLHL) superfamily with a C-terminal six-blade β-propeller domain, a *Plasmodium*-specific N-terminal domain, and a BTB/POZ domain [3,20]. Comparative structural analyses of the BTB domain of Pfk13 clusters with the BTBs of Potassium (K+) Channel Tetramerization Domain (KCTD) protein family exhibited the highest similarity to KCTD17 [32]. The KRP domain of Pfk13 showed a conserved and solvent-exposed shallow pocket that is like other KRP domains containing proteins [33]. 

The non-synonymous mutations, all present after positioning 440 amino acids in the propeller domain of the Pfk13 gene, and we herein using 337–726 amino acids residues for the heterologous expression and structural modelling. The recombinant plasmid was confirmed by double enzymatic assay and was afterwards transinfected with an SF9 cell; the SDS-PAGE showed that Pfk13-F446I and Pfk13-C580Y were found in precipitants and a little amount of the protein was visible in the elution, which might be explained why the corresponding signal/band in the elution fraction observed by WB was not obtained, as well as for the difference in purity. Given the reason that the expression profile would interfere with protein activity, we adopted various standardizations for conditions, either by lowering the induction temperature (low as 15 °C), altering the incubation time, or by changing another vector (PCEP4) and cell host (Expi293 cell); but this did not significantly alter the expression profile (data were not shown). We speculated that the conformational change from amino acid in the 446 and 580 sites could be misleading, because the Pfk13-F446I and Pfk13-C580Y mutations added 11 and 9 hydrogen bonds variations, respectively, when compared to WT. In addition, the yield of those two proteins of Pfk13-F446I and Pfk13-C580Y were 0.01 mg/mL, compared to the WT (0.02 mg/mL), also affected by the conformational change of the amino acid replacement by those two mutations. Similarly, the Gigaxonin mutation C464Y, located in the KREP central channel, such as C580Y in Pfk13, was also associated with decreased Gigaxonin protein abundance [34]. Therefore, we speculate that amino acid changes at the shallow pocket positions, which were predicted to be involved in substrate binding, are too functionally damaging to provide a long-term competitive advantage. However, a lot of Pfk13 ART-R alleles may alter other properties of Pfk13, such as its abundance, through altered protein synthesis, folding, or stability. Hence, more appropriate conditions for expression of those two proteins should be sought.

AlphaFold was confirmed to have predicted the protein structure modelling with high accuracy when compared to the experimental results in SARS-CoV-2, CYP102A1 (one of cytochrome P450 superfamily of enzymes), and KCTD proteins [35,36,37,38]. In this study, the Pfk13-F446I and Pfk13-C580Y mutations could lead to destabilization of Kelch domain structure by comparative structural analysis. Additionally, the C580 residue forms a hydrogen-bond with G533 and is predicted to lead to a steric clash with G533 and the loss of a hydrogen-bond, which was not found in Pfk13-WT, suggesting that molecular dynamics simulations was needed for further study, and that as more data become available this model will improve.

**Figure 5:**
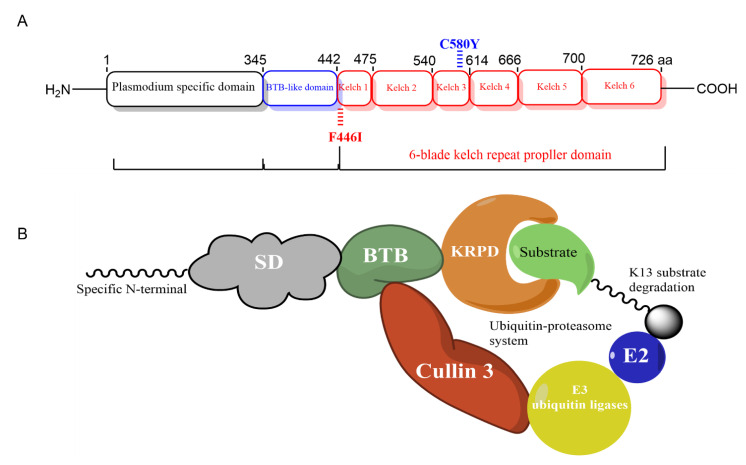
Mutations and structural representation of Pfk13 and its putative function as substrate adaptor. (**A**) Mutations in the Pfk13 protein involved in artemisinin resistance by WHO. The validated mutations F446I and C580Y were marked in the Kelch 1 and Kelch 3 propeller domains. (**B**) Putative function of pfK13. The 3 domains were annotated in web-based database: *P. falciparum* specific domain (SD), BTB and Kelch-repeat propeller domain (KRPD). The BTB domain is expected to bind a scaffold Cullin protein, subsequently with E3 ubiquitin ligases, while the KRPD likely binds to the substrate molecule(s) further ubiquitinated and possibly degraded by the ubiquitin-proteasome system. As shown for structural representation, Pfk13 was recognized as a monomer.

The results of in vivo and in vitro experiments showed that F446I and C580Y mutations were associated with ART in the field, resulting in a delay in the clearance of ACTs or an increase in the ring survival rate (RSA) [13,18] (Figure 5A). The BTB/POZ motif mediates protein binding and homo-dimerization [39] and is predicted to be involved in binding to cullin 3, the largest family of E3 ubiquitin ligases [40,41], with the downstream Pfk13 domain providing a substrate adaptor [42]. That is, the Pfk13 proteins partner with an E3 ligase to bind and orient specific substrates ready for polyubiquitination by an E2 ubiquitin-conjugating enzyme, which in turn leads to their degradation by the ubiquitin-proteasome system (Figure 5B). 

Overall, this study provided Pfk13-F446I and Pfk13-C580Y heterologous expression through a BEVS expression system in a SF9 cell. We also adopted a novel modelling framework with AlaphFold to make predictions as to the effects of resistance mutations by Pfk13-F446I and Pfk13-C580Y on the ability of ACTs.

## 5. Conclusions

In conclusion, this work first provided the two mutations, Pfk13-F446I and Pfk13-C580Y, which were strongly correlated and heterologous with ART resistance, successfully expressed in the SF9 cell through the BEVS system. We also confirmed that the hydrogen bond disappeared between C580 and C533 in the WT, suggesting that the hydrogen bond may have been lost during the mutation. Moreover, the Pfk13-F446I and Pfk13-C580Y mutation added 11 and 9 hydrogen bonds variation that may lead to conformational change of the protein structure. Future work should pay more attention to the binding characteristics of those mutations related to the KRPD pocket and their binding substrates, which will further clarify the structure function of Pfk13.

## Figures and Tables

**Table 1:** The elements variation, conformational location, and spatial distribution of the C533 position of pfK13 between the WT model (PDB 4YY8) and C580Y model by AlaphFold in this study.

Model Type	Elements	x	Y	z
WT (PDB 4yy8)	N	−17.97	−26.264	18.84
WT (PDB 4yy8)	C	−18.141	−24.822	18.834
WT (PDB 4yy8)	C	−19.235	−24.407	17.863
WT (PDB 4yy8)	O	−19.36	−24.961	16.775
**WT (PDB 4yy8) ^1^**	**H**	**−0.39**	**−3.488**	**−1.942**
Model C580Y1	N	−0.003	−4.254	−1.409
Model C580Y1	C	−0.698	−4.612	−0.171
Model C580Y1	C	−2.213	−4.631	−0.346
Model C580Y1	O	−2.805	−3.605	−0.643
Model C580Y2	N	−1.574	5.829	−8.52
Model C580Y2	C	−2.887	5.914	−7.874
Model C580Y2	C	−2.776	6.238	−6.389
Model C580Y2	O	−2.229	5.443	−5.637
Model C580Y3	N	4.815	−7.399	−0.246
Model C580Y3	C	3.858	−8.028	0.66
Model C580Y3	C	2.45	−7.528	0.367
Model C580Y3	O	2.2	−6.335	0.442
Model C580Y4	N	−2.534	5.28	−2.05
Model C580Y4	C	−3.805	5.291	−1.329
Model C580Y4	C	−3.581	4.962	0.139
Model C580Y4	O	−3.097	3.886	0.458
Model C580Y5	N	5.558	−3.18	3.363
Model C580Y5	C	4.469	−3.103	4.333
Model C580Y5	C	3.169	−3.571	3.697
Model C580Y5	O	2.703	−2.969	2.743

^1^ The hydrogen in bold was missed in the C580Y model in this study.

## Data Availability

The datasets generated during and/or analyzed during the current study can be find in the main text and the supplementary materials.

## Supplementary Materials

The following supporting information can be downloaded at: https://www.mdpi.com/article/10.3390/pathogens11111271/s1, Table S1: The amino acid replacements, structural variations of Pfk13-F446I comparing with 3D model of Pfk13 from the Research Collaboratory for Structural Bioinformatics Protein Data Bank (RCSB PDB; ID: 4YY8). Table S2: The amino acid replacements, structural variations of Pfk13-C580Y comparing with 3D model of Pfk13 from the Research Collaboratory for Structural Bioinformatics Protein Data Bank (RCSB PDB; ID: 4YY8).
